# Supplementary material for: Improving Mitochondrial Function in Skeletal Muscle Contributes to the Amelioration of Insulin Resistance by Nicotinamide Riboside
Source: Int J Mol Sci. 2023 Jun 12;24(12):10015. doi: 10.3390/ijms241210015 (PMC10297940; doi:10.3390/ijms241210015)
Supplement: Supplementary file 1 [file ijms-24-10015-s001.zip › ijms-2427204-supplementary.pdf]

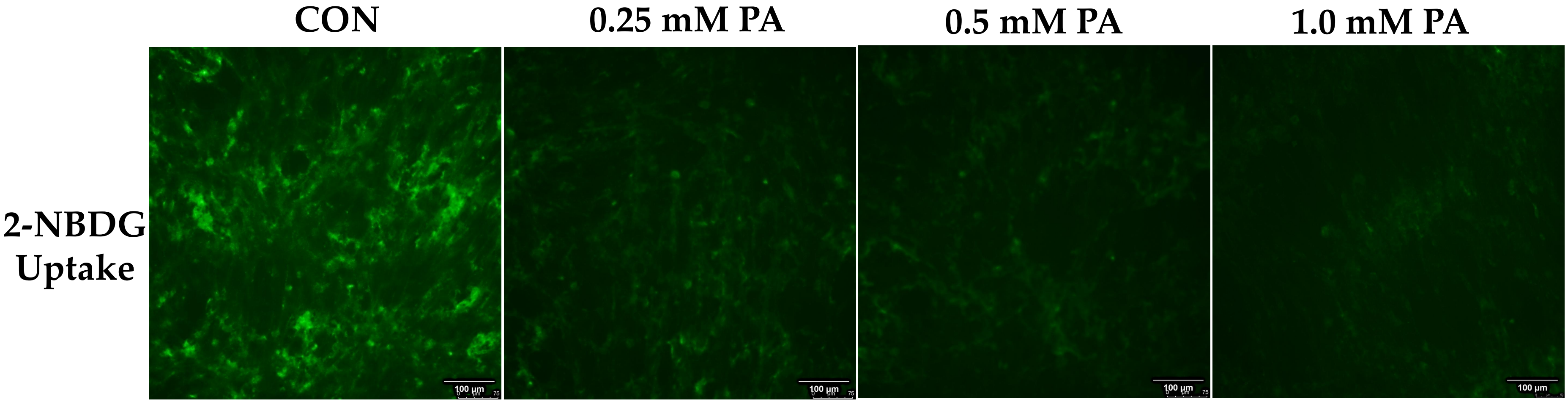

Figure S1. The dose–response experiments of PA in C2C12 myotube cells. Insulin stimulated uptake of 2-NBDG by C2C12 myotube cells treated with different dosages of PA for 24 h, the microscope magnification is 200×.
